# Expression profiling and transcriptional regulation of the *SRS* transcription factor gene family of common bean (*Phaseolus vulgaris*) in symbiosis with *Rhizobium etli*

**DOI:** 10.1371/journal.pone.0321784

**Published:** 2025-05-02

**Authors:** Litzy Ayra, Gladys Jiménez-Nopala, Gabriela Guerrero, Sara Isabel Fuentes, Alfonso Leija, Mario Ramírez, Georgina Hernández

**Affiliations:** Centro de Ciencias Genómicas, Universidad Nacional Autónoma de México, Cuernavaca, Morelos, México; Cornell University, UNITED STATES OF AMERICA

## Abstract

The SRS/STY transcription factors from the model legumes: *Lotus japonicus* and *Medicago truncatula*, are part of regulatory networks that play relevant roles for nodule development during the N-fixing symbiosis with rhizobia. In this work we analyzed the participation of the PvSRS transcription factors from common bean (*Phaseolus vulgaris)*, a most important legume crop, in the symbiosis with *Rhizobium etli*. Our phylogenetic analysis of SRS TFs across five plant species, including four legumes and *Arabidopsis thaliana,* identified clades that group SRS proteins that are highly expressed in legume nodules and in Arabidopsis roots. A qRT-PCR expression analysis of the 10 *PvSRS* in root/nodule of inoculated plants, revealed that all the *PvSRS* genes are expressed at different stages of the symbiosis, albeit at different levels. Based on what is known for *L. japonicus*, we demonstrated that the *PvSRS10* gene -with highest expression during symbiosis- is transcriptionally activated by NF-Y transcription factor, thus indicating its participation in the NIN-NF-Y regulatory cascade. Based on our previous work about the relevant role of members from the MADS-domain/AGL transcription factors as regulators of the N-fixing symbiosis, in this work we demonstrated the transcriptional regulation of *PvSRS10* by the MADS-TF PvFUL-like. Analysis of protein-protein interaction networks predicted thatPvSRS5 and PvSRS6 interact with proteins involved in transcriptional regulation and the auxin-activated signaling pathway. The regulatory mechanisms of PvSRS TF in common bean symbiosis may be related to auxin biosynthesis regulation, that is essential for determinate nodules development. Our study highlights the role of PvSRS TF in the N-/fixing symbiosis, a relevant process for sustainable agriculture.

## Introduction

The low nitrogen availability in agricultural soils is one of the principal factors limiting the productivity of economically important crops, including legumes, that are the main source of vegetable protein for the human and animal diet [1]. However, legumes can establish symbiosis with rhizobia bacteria that can capture atmospheric nitrogen to reduce it into ammonia, thereby supporting plant growth. The symbiotic nitrogen fixation (SNF) occurring in the legume-rhizobia association is the main source of nitrogen in the ecosystems. SNF is a complex process that begins with communication, via molecular signals, between the symbionts. The plant root releases flavonoids and isoflavonoids to the rhizosphere that are sensed by compatible rhizobia which in response secrete lipo-chito-oligosaccharides, commonly known as Nod Factors, that are recognized by specific receptors in the plant root hairs, triggering the rhizobia infection and the nodule organogenesis processes [2]. The infection responses start with the attachment of rhizobia to the legume root hairs, giving rise to several pre-infection responses including the invagination of plasma membrane that allows the formation and progress of infection threads through which the bacteria advance to the root cortex [3]. A local increase in sensitivity to cytokinin in the root cortex was proposed as being essential for the initiation of nodule primordia formation that will give rise to mature nodules [4,5]. Through endocytosis mechanism, rhizobia penetrate the cells of the nodule primordia forming structures known as symbiosomes in the mature nodules, where bacteria differentiate into bacteroid capable of carrying out the SNF [6].

The legume/rhizobia symbiosis process requires precise regulation of different signaling pathways, from each symbiont. Advances in legume genetics and genomics, especially during the last 20 years, have enhanced our understanding of legume genes relevant to SNF [7], providing a catalog with *ca.* 200 legume symbiotic genes that includes, among others, several transcription factors (TF) acting as global regulators with essential roles in the different stages of symbiosis. To this end, our work group has analyzed the role of different global regulators on the SNF of common bean (Ph*aseolus vulgaris*), the most important legume for human consumption in the world [8,9].

The *SHORT INTERNODES* (SHI) *RELATED SEQUENCES* (SRS) TFs family, also known as SHI or *STYLISH* (STY) [10], are exclusive to plants and are well-known for their role in various biological processes. Members of this family share two highly conserved domains: the Zinc finger, lateral root primordium type 1 (*Znf_LRP1*; IPR006510) which contain multiple finger-like protrusions that make tandem contact with their target molecule and the Short Internodes, C-terminal (*SHI_C*; IPR006511) that may act as a negative regulator of gibberellins responses through transcriptional control [11,12]. In Arabidopsis and other plants, SRS TF are involved in formation and development of different plant organs, such as: lateral roots, stem extension, leaf morphogenesis, pistil and stamen development, flowering time and in the regulation of phytohormone signaling [13].

SRS TF have been identified in some legume species and their participation in development and response to abiotic stress has been reported [14–19]. In soybean (*Glycine max*), 21 members of the SRS family were identified. RNA/seq data from SoyBase indicated that SRS TF are expressed in different plant organs: leaf, flower, pod, seed, root and nodule, being *GmSRS2, 9, 14* and *21* the most highly expressed in nodules [15]. Expression and functional analysis indicated that GmSRS TF play important roles in abiotic stress responses such as: drought and salinity [16]. Yang et al. (2021) identified 27 SRS genes from alfalfa (*Medicago sativa*); their structural and functional analysis revealed the response of *MsSRS* genes to salt and cold abiotic stresses. Similar analyses of white sweet clover (*Melilotus albus*) identified nine *MaSRS* genes. Their expression patterns in *M. albus* leaves showed differential expressions in stress treatments such as: salinity, low temperature, salicylic acid and methyl jasmonate [18]. Buyuk et al. (2022) identified 10 *PvSRS* genes that, according to RNA/seq data, are expressed in different tissues including nodules. The expression level of *PvSRS* genes was comparatively analyzed in two common bean cultivars, native from Turkey, with contrasting performance (resistance vs susceptibility) to salt stress, support the participation of PvSRS TF in this abiotic stress [19].

Contrasting to above mentioned studies that lack a solid analysis of the participation of SRS TF in the legume-rhizobia SNF, a detailed analysis of *Lotus japonicus* SRS TF evidenced their crucial role for nodule development in the SNF with *Mesorhizobium loti* [15]. The requirement of LjSRS TF for nodule emergence is attributed to a *LjNF-Y* dependent regulatory cascade, comprising *STY* genes and their downstream targets, *YUCCA1* and *YUCCA11*, involved in a local auxin biosynthesis at the post-initial cell division stage [15]. In addition, [14] provided evidence of the operation of an analogous MtNF-Y/MtSTY regulatory module, required for the indeterminate nodule patterning, in the *Medicago truncatula* N-fixing symbiosis.

Transcriptomic data from common bean *Negro jamapa* cv, from different plant tissues at various developmental stages, showed that most *PvSRS* genes are expressed in inoculated root and nodules of plants inoculated with *Rhizobium tropici* [20]. In this work, we further analyzed the participation of common bean PvSRS TF in the N-fixing symbiosis. We performed a phylogenetic analysis of SRS TF from common beans, other three legume species and Arabidopsis. Our analysis of the expression levels of each *PvSRS* gene at different stages of symbiosis of common bean (BAT93 genotype) with *R etli* CE3 indicated that every *PvSRS* gene is expressed in inoculated roots and nodules, albeit at different levels. *PvSRS5, PvSRS6*, and *PvSRS10* showed higher expression in these symbiotic tissues.

Like Lotus [15,21], our data on identification of *cis*- regulating elements in the promoter region as well as experimental evidence of *in planta* transcriptional activation, the *PvSRS10* gene is a direct target of PvNFY TF. We investigated if the *PvFUL*-like gene, from the MADS domain/ AGL TF family, previously evidenced as a relevant regulator of the common bean N-fixing symbiosis [22] is a transcriptional activator of the *PvSRS10* gene. Our bioinformatics and experimental analysis showed positive results, thus proposing the PvFUL-like TF as a transcriptional regulator of *PvSRS10*. Furthermore, a bioinformatic analysis of the PvSRS5 and PvSRS6 protein-protein interactions revealed their association with various TFs including Auxin Response Factors (ARFs) and auxin-induced proteins. Our results prompt us to propose that a similar regulatory cascade as that reported *L. japonicus* [15], is occurring in the common bean nodulation process: NF-Y and, in this case, also PvAGL TF activate the transcription of *PvSRS* genes that may interact with other TFs to regulate the auxin synthesis for nodule development.

## Materials and methods

### Phylogenetic analysis

For the phylogenetic analysis the *SRS/SHI/STY* genes previously reported *for Arabidopsis thaliana* [23]*, Glycine max* [16], *Medicago truncatula* [10], *Lotus japonicus* [15] and *Phaseolus vulgaris* [19] were included.

Gene sequences translated protein sequences and RNA-seq gene expression data of *SRS* TFs for each plant species were retrieved from the following databases. For *A. thaliana*: The Arabidopsis Information Resource TAIR (https://www.arabidopsis.org); for *P. vulgaris*: the Gene Expression Atlas (GEA), (Pv GEA (https://www.zhaolab.org/PvGEA/) [20]; for *G. max* the ePlant BAR page (http://bar.utoronto.ca/eplant_soybean/) [24], for *M. truncatula* (https://phytozome-next.jgi.doe.gov/) and for *L. japonicus* the Lotus Base (https://lotus.au.dk/).

The protein sequences from *SRS* genes were analyzed in InterPro (http://www.ebi.ac.uk/interpro/) to confirm the presence of two characteristic domains from this gene family: *Znf_LRP1* and *SHI_C* [11,12]. The whole set of 60 SRS protein sequences from Arabidopsis and four legume species used for phylogenetic analysis are listed in (S1 Table). MUSCLE [25] was used for multiple sequence alignment of the given protein sequences; the alignment was used to construct the phylogenetic tree by MEGA 7 software [26] and the neighbor-joining method with the bootstrap test replicated 1,000 times. Finally, Interactive Life Tree (iTOL, https://itol.embl.de/index.shtml/) was used for the phylogenetic tree visualization.

### Plant material, growth conditions and bacterial strains

The common bean (*P. vulgaris*) Mesoamerican cv BAT93 was used in this work [27]. Seeds were surface sterilized and germinated as previously reported [22]. Germinated seedlings were planted in pots with wet sterile perlite and were grown in growth chambers under controlled environmental conditions (25–28°C, 16 h photoperiod, 60% humidity). For SNF conditions, plantlets were inoculated with 1 ml saturated liquid culture of *R. etli* CE3 per plant and were watered every 3 days with N-free B&D nutrient solution [28]. For non-inoculated conditions, a full nutrient B&D solution (5 mM N-content) was used to water the plants. Common bean composite plants with transgenic roots were generated through *Agrobacterium rhizogenes* K599 genetic transformation, as described below. The growth conditions of composite plants were like those described for wild-type plants.

*Nicotiana benthamiana* L. plants, used for *PvSRS10* promoter expression analysis, were grown in pots with peat moss: vermiculite (1:3), under greenhouse conditions at 16h photoperiod and watered with B & D nutrient solution [28]. *Agrobacterium tumefaciens* strain GV3101 was used for co-infiltration of *N. benthamiana* leaves.

### RNA isolation and quantitative RT-PCR analysis

Total RNA was isolated from frozen tissues collected directly into liquid nitrogen and stored at −80°C using the Plant/Fungi Total RNA Purification Kit according to the manufacturer’s instructions (Norgen Biotek Corp., Thorold, ON, Canada). Total RNA was quantified using the Nanodrop spectrophotometer (Thermo Fischer Scientific, Inc., Waltham, MA, USA). For quantification of transcripts, total RNA (1 µg) was treated with DNaseI RNase-free (Thermo Fischer Scientific, Inc., Waltham, MA, USA) to remove genomic DNA. First, strand cDNA was synthesized using Revert Aid H Minus First Strand cDNA Synthesis Kit (Thermo Fisher Scientific, Inc., Waltham, MA, USA). The resulting cDNAs were then diluted (1:40) and used to perform qRT-PCR assays using SYBR Green PCR Master Mix (2X) (Thermo Fischer Scientific, Inc., Waltham, MA, USA), following the manufacturer’s instructions. The sequences of oligonucleotide primers used for qRT-PCR of each gene and for the three housekeeping genes HSP, MDH and UBQ9 [29] are provided at (S2 Table). Assays were run in 96-well plates using the 7300 Real-Time PCR System and 7300 System Software (Applied Biosystems, Foster City, CA, USA) with settings of 50^°^C for 2 min, 95°C for 10 min, and 40 cycles of 95°C for 15 s and 57°C for 60 s. The efficiency of the RT-qPCRs was confirmed using a standard curve for each primer evaluated. Finally, the relative expression was calculated by the “comparative Ct method”. Student’s *t*-test was performed to evaluate the significance of the differential expression using the mean values from three biological replicates and three technical replicates for each condition, using the GraphPad Prismv8.0 for Windows (GraphPad Software, San Diego, CA, USA).

### Identification of putative *cis*-regulatory elements in promoter regions

Up to 4 Kb of the DNA sequence from the promoter region, immediately upstream of the initiation codon of each analyzed gene, was retrieved from Phytozome v12.1 (https://phytozome.jgi.doe.gov/pz/portal.html). The Clover (Cis-eLement OVERrepresentation) tool (https://bu.wenglab.org/clover/) [30], was used to identify over-represented significant motifs in each DNA sequence, using precompiled JASPAR (https://jaspar.elixir.no/) motifs. Clover analyses were conducted using default parameters.

The enrichment of AGL or NF-YA transcription factors binding sites was analyzed for the promoter region of each *PvSRS* gene.

The raw score represents the probability that the motif incidence score is equal to or greater than the user-defined threshold of 6. A *p*-value threshold of 0.05 was applied. Therefore, each motif with a raw score above 6 having a *p*-value below 0.05 are considered statistically overrepresented [30].

### Plasmid construction and evaluation of transcriptional activation of *PvSRS10* by agroinfiltration of tobacco leaves

To evaluate the transcriptional activation of the *PvSRS10* (Phvul. 011G206400) promoter activity, one reporter and two effector plasmids were constructed. For the reporter plasmid, bearing the *PvSRS10* gene promoter fused to the GPF/GUS fused reporter genes, a 2,001 bp DNA fragment upstream of the *PvSRS10* start codon, was amplified from *P. vulgaris* genomic DNA using the specific primers Up-*pPvSRS10* 5´- CACCTTTTAAGTGAGCCAAAT and Lw-*pPvSRS10* 5´- TGGAACAACAACAAGGTTTT -3´. The amplified fragment was then cloned into the pENTR/D-TOPO (Thermo Fisher Scientific, Inc., Waltham, MA, USA) vector. The pENTR-pSRS10 plasmid was subsequently recombined by Gateway® LR Clonase™ II Enzyme Mix (Invitrogen, Waltham, MA, USA) into the pBGWFS7 destination vector containing the DNA fragment coding for green fluorescent protein (GFP)/b-glucuronidase (GUS) [31]. The resulting reporter plasmid (pSRS10/GFP) contained the *PvSRS10* promoter upstream of the *GUS-GFP* gene fusion.

For the effector construct with overexpression of TF *PvNF-YA1* gene (Phvul.001G196800), its coding sequence (CDS) was PCR-amplified using as template cDNA from common bean roots and the specific primers Up-*PvNF-YA1* 5´- CACCATGGCGATGCA-3´ and Lw-*PvNF-YA1* 5´- TTGATTCTTTGCTAGTCAAACTTT-3´ and the purified PCR product (993 bp) was cloned into the pENTR/D-TOPO vector. Subsequently the *PvNF-YA1* CDS region was recombined by Gateway system into the pTDTO plasmid bearing the 35S cauliflower mosaic virus (35SCaMV) promoter and the *tdTomato* (red fluorescent protein) reporter gene [32]. The resulting effector vector, OE/NF-YA1, contained the 35S promoter upstream of the PvNF-YA1/tdTomato chimeric gene. The effector plasmid with overexpression of the TF *MADS-box* gene: *PvFUL*-like (Phvul.008G027800) from the 35S promoter (OE/FUL), was previously reported [22]. Every construct was verified by DNA sequencing.

The *Nicotiana benthamiana* leaves infiltration method [33], was used for the evaluation of the transcriptional regulation of *PvSRS10* gene. The empty vector (EV) used for plasmids construction (pTDTO) as well as the pSRS10/GFP, RFP_OE/NF-YA; RFP_OE/FUL were introduced, by electroporation, into *Agrobacterium tumefaciens* strain GV3101. For co/infiltration the resulting *A. tumefaciens* strains were used in a 1:1 ratio in 4-week-old *N. benthamiana* leaves. Five days after infiltration, the transiently transformed *N. benthamiana* leaves were inspected by inverted confocal laser scanning microscope FV1000 (with a 40_/NA 0.75 dry objective) using the following conditions (Excitation λ= 488 nm, Emission λ= 520 nm). The images were processed using Image J analysis software (Fiji Is Just Image J1), [34].

### Plant transformation and generation of composite plants

The plasmids for overexpressing (OE/FUL) or silencing (RNAi_AGL) TF *PvAGL* genes, as well as the EV used for these constructions, have been reported [22]. These plasmids were introduced by electroporation into *Agrobacterium rhizogenes* K599, which was then used for plant transformation as described [35] with minor modifications [32]. In addition, the presence of red fluorescence from the tdTomato reporter gene was routinely checked in the putative transgenic roots/nodules using a fluorescence stereomicroscope.

### Protein interaction network

Protein-protein interactions (PPIs) were analyzed using STRING database (http://string-db.org/) [36]. The analysis was performed to identify and predict functional associations between proteins. The target proteins, PvSRS5 and PvSRS6, were selected based on its high expression in nodules in early stages of symbiosis, which is related to greater auxin synthesis and cell division that guarantees the development of nodules and colonization by rhizobia.

### Statistical analyses

The graphs were generated using the ggplot2 library with RStudio (v1.4.1106) or GraphPad Prism 9. The specific statistical tests performed are indicated in the legend of the corresponding figures. Raw data used for statistical analysis for Figs 2 and 4 are provided in two separate sheets of S3 Table.

## Results

### Evolutionary relationships of the SRS TFs in Arabidopsis and legume plants

**Fig 1:**
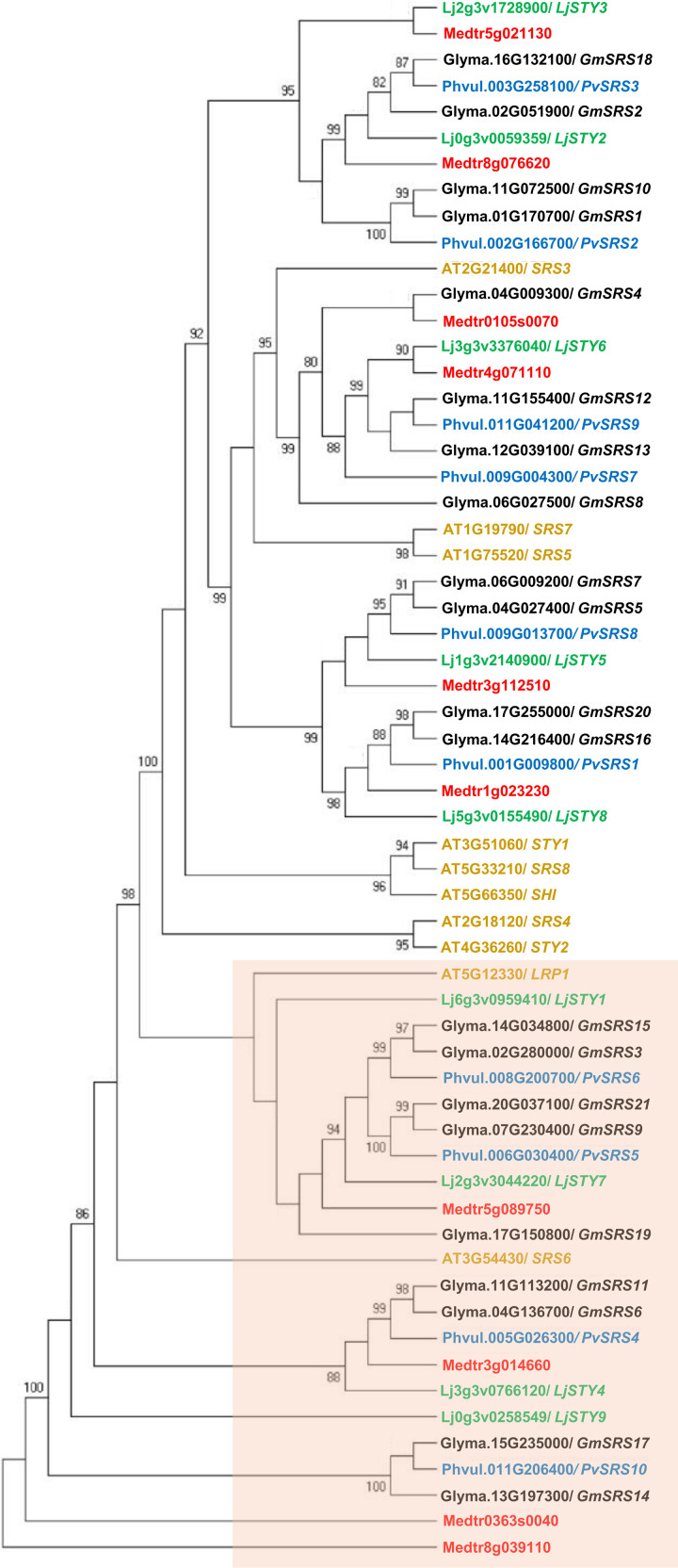
The phylogenetic relationship between 60 SRS proteins from *Phaseolus vulgaris* (10, blue), *Glycine max* (21, black), *Medicago truncatula* (10, red), *Lotus japonicus* (9, green) and *Arabidopsis thaliana* (10, ocher). The unrooted tree was constructed using full-length protein sequences that are reported in S1 Table. Bootstrap values greater than 80 are presented at the corresponding node. The pink box highlights clades grouping genes from *Arabidopsis* and legumes, that share high expression in roots or in roots and nodules.

The SRS TF family is only present in plants and the number of family members varies in different plant species. In this work we carried out a phylogenetic analysis of SRS TFs from 5 different plant species, including the model plant *Arabidopsis thaliana* (Arabidopsis) and 4 legume plants. The latter included the two model legumes: *Lotus japonicus* (Lotus) and *Medicago truncatula* (Medicago), *Phaseolus vulgaris* (common bean) which is the species analyzed in this work and the most important legume for human consumption worldwide [8,9] and *Glycine max* (soybean), a very important legume crop closely related to common bean [37]. The protein sequences encoded by *SRS* genes were previously identified, but for our analysis we verified each protein for the presence of the two characteristic domains from this gene family: the *Znf_LRP1* and *SHI_C* domains. On this basis, we excluded from our analysis the proteins that lack the characteristic domains, encoded by AT1G32730 (*AtSRS11*) gene from Arabidopsis and by Medtr4g099070 gene from Medicago [13,16]. Consequently, for the maximum likelihood phylogenetic tree depicted in (Fig 1) we analyzed a set of 60 SRS protein sequences (S1 Table) that included 10 SRS proteins for Arabidopsis [23], 9 for Lotus [15], 10 for Medicago [13], 21 for soybean [16] and 10 for common bean [19].

Clades that interested us, because of the objective of this work, are highlighted in (Fig 1). These include two Arabidopsis proteins from the SRS TF family (LPR1 and SRS6) and several legume SRS proteins. LRP1 is highly expressed in all stages of lateral root development, participating in auxin response modules to regulate lateral root development and auxin homeostasis in Arabidopsis [38]. Interestingly, legume genes grouped in these clades are known to be highly expressed during root and nodule development, mainly *LjSTY4*, *LjSTY7*, and *LjSTY9* [15], *GmSRS9*, *GmSRS14*, and *GmSRS21* [16], as well as *Medtr3g014660* and *Medtr5g089750* (https://bar.utoronto.ca/eplant_medicago/). The SRS

proteins from Medicago and Arabidopsis, lacking characteristic SRS domains, that we excluded from our phylogeny did not group in root/nodule clade highlighted in Fig 1.

Contrastingly, other Arabidopsis genes not associated with these clades have acquired different functions. For example, At3G51060/*STY1* and *At1G19790*/*SRS7*, show their highest expression during flower development, regulating the development of specific tissues such as the stigma and carpels [39].

### Expression analysis of *PvSRS* genes in root and nodules of SNF plants

Transcriptomic data from *P. vulgaris* cv. *Negro jamapa* inoculated with *Rhizobium tropici* [20] revealed that all *PvSRS* genes are expressed in inoculated roots and nodules, though at varying levels. In this study, we carried out a qRT-PCR analysis to evaluate the expression level of each *PvSRS* gene in roots and nodules of common bean BAT93 plants inoculated with *R. etli* CE3 at different stages of symbiosis as compared to uninoculated or N-fertilized roots. We followed the *PvSRS* gene nomenclature previously reported [19].

**Fig 2:**
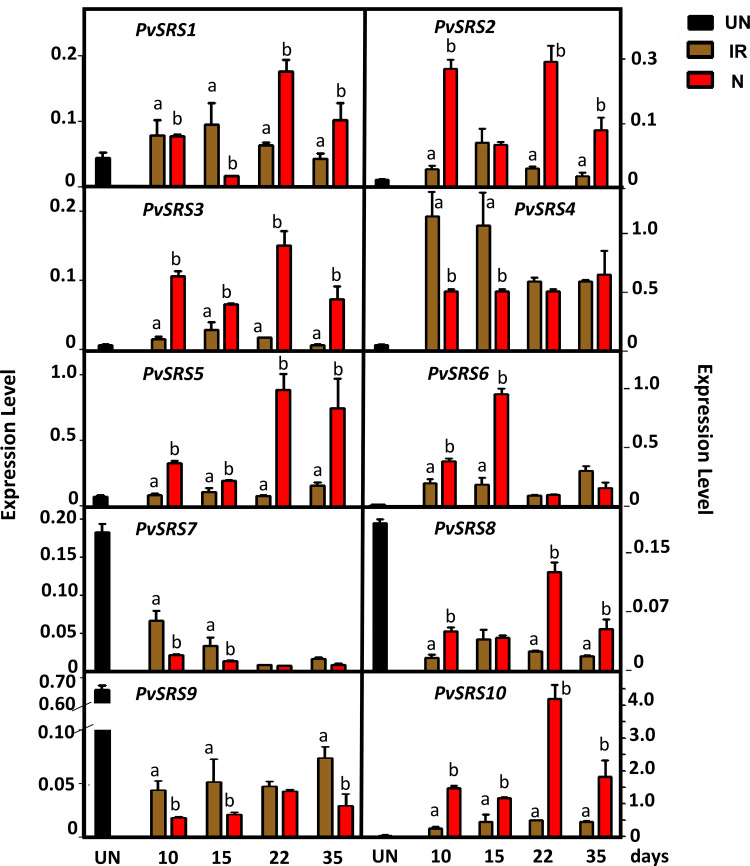
Expression analysis of common bean *PvSRS* genes in roots and nodules. Expression levels were determined in uninoculated (UN) roots (black) collected 3 days after germination and in inoculated roots (brown), or nodules (red) harvested at the indicated time points, corresponding to days post-inoculation of *P. vulgaris* BAT93 plants inoculated with *R. etli*. Expression levels refer to gene expression, based on the Ct value, normalized with the expression of housekeeping genes. (*HSP*, *MDH* and *UBQ9*). The name assigned to each *P. vulgaris* SRS gene corresponded to those from [19]. The gene IDs are shown in Supplementary S1 Table. Values from each timepoint for each gene were statistically analyzed by Student’s *t*-test. In each case, different lowercase letters indicate significantly different values from roots vs. nodules (*p*-value < 0.05).

As shown in (Fig 2), all *PvSRS* genes are expressed in inoculated roots and nodules, albeit at different levels. Notably *PvSRS4*, *PvSRS5*, *PvSRS6* and *PvSRS10* exhibited higher

expression levels in inoculated roots and nodules compared to the extremely low level in uninoculated roots (Fig 2). *PvSRS1*, *PvSRS2*, *PvSRS3* and *PvSRS8* showed lower expression levels in inoculated roots and nodules, ranging from 2-fold to 26-fold lower as compared to the highly expressed genes. *PvSRS7* and *PvSRS9* showed much higher expression level in uninoculated roots, thus suggesting their relevant participation in root development rather than in SNF (Fig 2). *PvSRS4* expression was highest in inoculated roots at early symbiotic stages (10–15 dpi) (Fig 2). *PvRSR6* was highly expressed in immature (10–15 dpi) nodules (Fig 2). *PvSRS2* and *PvSRS3* showed high expression in both mature and immature nodules, *PvSRS8* and *PvSRS10* showed higher expression in mature nodules (22 dpi) and *PvSRS5* showed its highest expression in mature and senescent nodules (22–35 dpi) (Fig 2). These results suggest potentially important regulatory roles of PvRSR5 and PvSRS6 during early nodule development and PvSRS5 also in mature nodules (Fig 2). *PvSRS10* reaches the highest expression level in inoculate roots and nodules as compared to every other *PvSRS* gene, thus pointing to its relevant participation in the symbiotic process (Fig 2). These results point to the participation of all the members of PvSRS TF family in different stages of the common bean N-fixing symbiosis, perhaps except for PvSRS7 and PvSRS9.

Our qRT-PCR expression level data (Fig 2) are consistent with those previously reported [19] obtained from RNA-seq analysis (S1 Fig). The slight differences observed between two sets of data (S1 Fig) may be due to a different common bean genotype used in each study and the developmental state of nodules (days post inoculation), something that was not informed in the previous work [19].

### Transcriptional activation of *PvSRS10* gene by NF-Y TF

As previously shown for nodule development of Lotus [15,21], the regulation of STY TF is associated with an NF-Y-dependent regulatory cascade. In this cascade, *STY* genes are direct targets of NF-Y TF and, in turn, activate the transcription of *YUCCA1* and *YUCCA11*, that are responsible for local auxin biosynthesis during the cell division phase. In this work we investigated if, similarly, the common bean *PvSRS* genes transcription is activated by NF-Y TF.

First, we looked for NF-Y TF binding sites, the so-called CCAAT box [40], in the promoter region of every *PvSRS* gene. Using the Clover tool [30] we identified significantly over-represented motifs for NF-Y TF binding distributed across every *PvSRS* gene promoter, appearing from 1 to 6 times in each gene (S4 Table). The *PvSRS3*, *PvSRS5* and *PvSRS10* gene promoters showed a higher frequency of NF-Y TF binding motifs (S4 Table). These *PvSRS* genes showed a similar expression profile with the highest expression in mature nodules, though the expression level of *PvSRS3* was lower (Fig 2).

The significant over-representation of motifs recognized by NF-Y TF in every *PvRSR* gene promoter (S4 Table), prompted us to experimentally demonstrate *in planta* transcriptional activation of *PvSRS* by PvNF-Y TF. For this, we selected the *PvSRS10* gene, that showed the highest expression in roots and nodules (Fig 2) and the system of agroinfiltration of *Nicotiana benthamiana* leaves to evaluate transient gene expression [33]. This system has been widely used for the *in planta* transient expression of heterologous genes and gene product detection. As a higher plant *N. benthamiana* shares common cellular compartmentalization, cofactors and coenzymes with other plants, thereby easily enabling heterologous plant gene expression and protein function [41].

To promote transcriptional activation/repression the plant NF-Y TF act as heterotrimers (NF-YA/B/C) [42,43]. The NF-YA subunits from different plant species have high primary sequence homology [44]. The NF-YA subunit is localized to nucleus, and it has a domain with two important regions: one for interaction with the other NF-Y subunits and another for DNA-binding that recognizes CCAAT-box from promoters of target genes [44]. Whereas in animals each NF-Y subunit is encoded by a single gene, structural and functional diversification has occurred in plants, leading to the emergence of gene families comprising between eight and 39 members for each subunit [44]. In Medicago, the NF-YA1/2, NF-YB16/18 and NY-YC1/2 subunits are highly expressed in nodule tissues and regulate the expression of relevant symbiotic genes [45]. Boudin et al., (2015) clearly demonstrated the interaction of NF-YA, B and C symbiotic subunits by co-expressing their coding genes in *N. benthamiana* leaves and using co-immunoprecipitation and bimolecular fluorescence complementation approaches to identify the transiently expressed proteins and their interaction. In addition, the formation of stable NF-Y trimer with common bean PvNF/Y subunits, encoded by *PvNF-YA1*, *PvNF-YB7* and *PvNF-YC1* that are orthologs of Medicago symbiotic *NF-Y*s and participate in SNF [46], was demonstrated [45]. Previous work has shown that the interaction specificity between NF-YA, B and C subunits is weak both in Arabidopsis [43,45,47,48] and in Medicago where, with the exception of two subunits, all the MtNF-YB subunits can interact with MtNF-YC1 and MtNF-YC2 *in planta*, something that is independent of their sequence similarity or their expression in different paly tissues [45].

The current model for assembling of the heterotrimeric NF-Y complex proposes that the other two subunits (NF-YB and NF-YC) interact in the cytoplasm and subsequently the heterodimer is translocated into the nucleus where it couples with the NF-YA subunit to form the heterotrimeric protein [49]. The subcellular localization of Medicago NF-Y subunits expressed in *N. benthamiana* revealed the strict localization of MtNF-YA1/2 to the nucleus while MtNF-YB16 and MtNF-YC1/2 was localized to both the nucleus and the cytoplasm [45].

On this basis, to test the proposed transcriptional activation of *PvSRS10* by PvNF-Y TF we co-infiltrated *N. benthamiana* leaves with an effector vector overexpressing the *PvNF-YA1* together with a reporter vector bearing a chimeric gene with the *PvSRS10* promoter fused to the GFP reporter gene, as well as appropriate controls. *N. benthamiana* leaves were examined by confocal microscopy for the observation of synthesized reporter fluorescent proteins, product of transient expression of the reporter gene.

**Fig 3:**
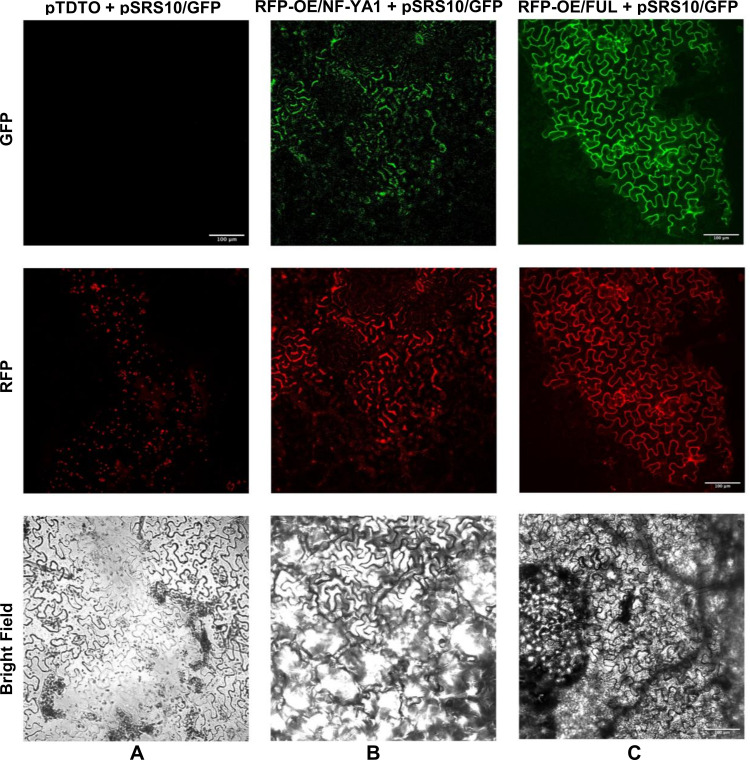
Transcriptional activation of *PvSRS10* gene promoter by PvNF-YA1 and PvFUL (AGL) TFs. *Nicotiana benthamiana* leaves were infiltrated with *Agrobacterium tumefaciens* carrying different constructs and, after five days, these were analyzed by confocal microscopy using the green fluorescence channel (upper panels), the red fluorescent channel (middle panels) or the bright field (lower panels). **A**: Co-infiltration of pTDTO (empty vector) plus the reporter plasmid: pSRS10/GFP. **B**: Co-infiltration of the effector plasmid: RFP_OE/NF-YA1 plus the reporter plasmid: pSRS10/GFP. **C**: Co-infiltration of the effector plasmid RFP_OE/FUL plus the reporter plasmid: pSRS10/GFP.

The efficiency of the technique used was confirmed by visualizing the red fluorescence in *N. benthamiana* leaves agroinfiltrated with only the pTDTO empty vector S2A Fig or each one of the effector plasmids S2B and S2C Fig. In each case the red fluorescence, derived from the constitutive expression of the tDTomato gene present in the plasmid backbone, was clearly observed. Similarly, only the red fluorescence was visible in leaves co-infiltrated with each of the effector plasmids plus the empty vector pBGWFS7, bearing the GFP without any cloned promoter S2D and S2E Fig. In addition, leaves infiltrated with only the empty vector pBGWFS7 S2F Fig or only the reporter plasmid S2G Fig, showed no fluorescence, thus indicating that the Pv*SRS10* gene promoter was not expressed by endogenous TF. Leaves co-infiltrated with the empty vector pTDTO plus the reporter plasmid only showed red fluorescence and no green fluorescence was visible (Fig 3A).

Contrastingly, the leaves co-infiltrated with the reporter plus the effector plasmid overexpressing *PvNF-YA1* showed clear green fluorescence (Fig 3B). Our interpretation of this result is that, in co-infiltrated leaves, the heterologous NF-YB and NF-YC subunits from *N. benthamiana* interacted and functioned effectively with the PvNF-YA subunit. Recent transcriptomic data from different tissues of *N. benthamiana* plants [50] show the high level of gene expression of 3 *NbNF-YB* genes and 3 *NbNF-YC* genes in mature leaves (S5 Table), something that supports our interpretation.

### Does PvFUL-like TF, from the MADS-Domain/AGL family, activate transcription of the *PvSRS10* gene?

Previous work from our group [22] showed the relevant participation of common bean MADS/AGL TF in the regulation of the N-fixing symbiosis with rhizobia. This is a large TF family that controls the development of almost every plant organ in a variety of plant species. The common bean genome encodes for 93 *PvAGL* genes, 16 of these are expressed in roots and nodules, being the *PvFUL*-like gene – orthologous to the *AGL8* or *FUL* gene from Arabidopsis [51] - the one with highest expression during symbiosis. Phylogenetic analysis revealed clades that grouped the AGL proteins highly expressed in Arabidopsis roots and in legume roots and nodules [22]. Using reverse genetic approach: symbiosis phenotypic analysis of common bean plants with transgenic root/nodules modulated -silencing or over-expression- in *PvFUL*-like expression level, we demonstrated the participation of this TF in root architecture, rhizobial symbiotic infection, the expression of known early symbiotic genes, nodulation, nitrogenase activity in nodules and the AON (Autoregulation Of Nodulation) process [22]. Since in our previous work [22] we did not identify transcriptional target symbiotic genes for PvAGL TF, our current research includes projects aiming to identify such targets and their participation in symbiotic regulatory cascades. On this basis, one of the objectives of the present work was to investigate if the *PvSRS* genes that are expressed during the SNF (Fig 2), are transcriptionally regulated by PvAGL TF, besides their regulation by NF/Y TFs.

On this basis, using the Clover tool [30], we searched for significantly over-represented motifs for AGL TF binding, the so-called CArG box [52,53], in the promoter region of each *PvSRS* gene. Notably, we did find over-represented motifs for AGL TF distributed across every *PvSRS* gene, appearing from 4 to 14 times in each gene promoter (S4 Table). The *PvSRS4*, *PvSRS8* and *PvSRS10* gene promoters showed a higher frequency of AGL TF recognized motifs. As previously shown (Fig 2) *PvRSR10* showed the highest expression level in mature nodules, similar as PvSRS8, while PvSRS4 showed the highest expression in inoculated roots and high expression in young, mature and senescent nodules.

These results prompted us to experimentally analyze if the PvFUL-like TF, which showed the highest expression in inoculated roots/nodules [22] as transcriptional activator of *PvSRS10*. For this we used a similar system as for the PvNF/Y TF analysis based on the co-infiltration of *N. benthamiana* leaves with an effector vector overexpressing *PvFUL-like* and a reporter vector bearing the chimeric gene with the *PvFUL-like* promoter fused to the GFP reporter gene, as well as appropriate controls. The *N. benthamiana* leaves co-infiltrated with the effector vector over-expressing *PvFUL-*like plus the reporter plasmid clearly showed green fluorescence (Fig 3C). This result proposes the PvFUL-like (AGL) TF as a novel transcriptional activator of *PvSRS10.*

**Fig 4:**
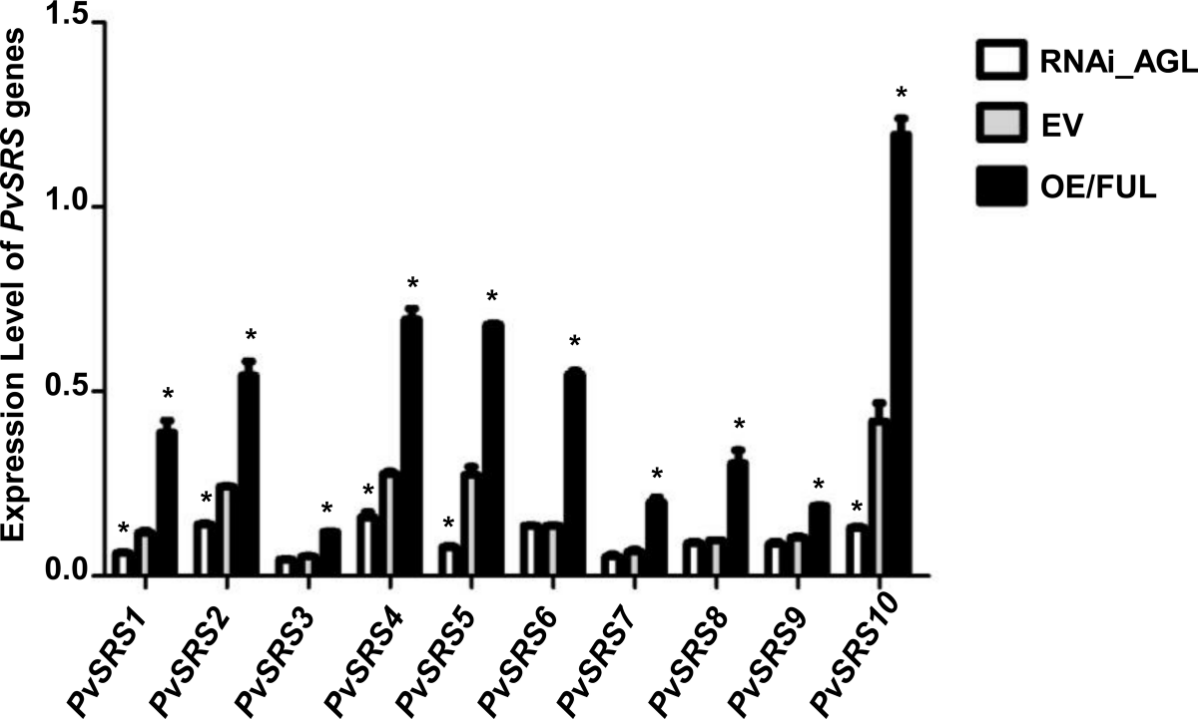
Expression level of *PvSRS* genes in transgenic nodules expressing the RNAi/AGL or OE/FUL constructs as compared with EV. The *PvSRS* genes expression was evaluated at 22 dpi. Expression level refers to genes expression, based on Ct value, normalized with the expression of three housekeeping genes (HSP, MDH and UBQ9). Asterisks indicate significant difference between silenced or overexpressing transgenic nodules with respect to EV at each time point analyzed by Student´s *t*- test (p value < 0.01). Data showed were obtained from three biological replicates, and one technical replicate each from common bean plants.

To reinforce this conclusion, we analyzed the expression level of *PvSRS genes* in transgenic nodules of *R. etli*-inoculated composite bean plants, with modulated expression of *PvFUL-like* overexpression. As compared to nodules transformed with the EV, the nodules expressing the *RNAi/FUL* construct showed 80% of gene silencing and those expressing the *OE/FUL* construct showed 8-fold *PvFUL* expression level. The expression level of each *PvSRS* gene was determined in transgenic nodules at 22 dpi, modulated in *PvFUL-like* expression; results are shown in Fig 4. Nodules over-expressing *PvFUL*-like gene showed increased expression level of every *PvSRS* gene, these were 1.8 to 4-fold higher than control (EV) nodules. In silenced (RNAi/FUL) nodules, a significantly decreased expression level was observed for *PvSRS1*, *PvSRS2*, *PvSRS4*, *PvSRS5* and *PvSRS10* genes, that showed 28% to 57% expression level as compared to the value form each gene in EV nodules. Our results (Fig 4) agree with those from (Fig 3) evidencing PvFUL-like as a transcriptional activator of *PvSRS* genes.

### Predicted protein-protein interaction network for PvSRS5, PvSRS6

The functional relationships of the PvSRS5 and PvSRS6 proteins, which are highly expressed during the early stages of nodule development (Fig 2), were predicted using the STRING protein interaction database [36].

Results from the functional protein association network of PvSRS5 revealed the number of 10 edges, an average local clustering coefficient of 0.597, and a protein-protein interaction (PPI) enrichment p-value of 9.34e-05. The predicted interacting proteins were linked with three significantly enriched biological processes GO (Gene Onthology) terms: auxin-activated signaling pathway (GO:0009734), regulation of transcription, DNA-templated (GO:0006355), and regulation of cellular processes (GO:0050794) and to two significantly enriched molecular function GO terms: DNA-binding transcription factor activity (GO:0003700) and DNA binding (GO:0003677).

In the PvSRS6 network, the number of edges was also 10, with an average local clustering coefficient of 0.6 and a PPI enrichment p-value of 4.03e-06. Three of the predicted interacting proteins were linked to the biological processes GO terms: regulation of transcription, DNA -template (GO:0006355) although this was not significantly enriched.

**Fig 5:**
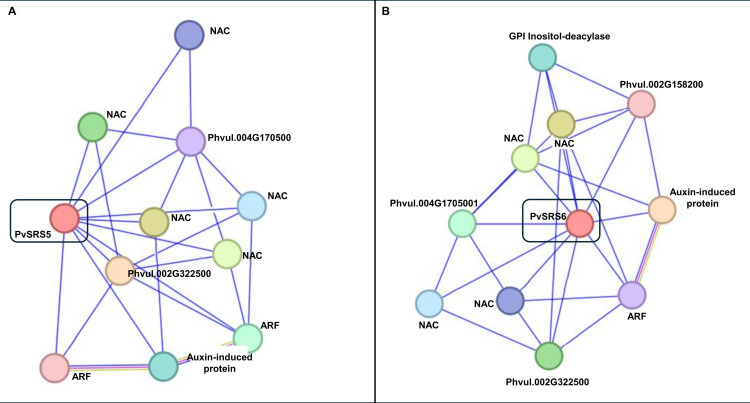
Protein-protein interactions network diagram for PvSRS5 (A) and PvSRS6 (B) predicted using the STRING database. Each colored circle represents a protein that potentially interacts with PvSRS5 (A) or PvSRS6 (B) and with other proteins from the diagram. For proteins with non-characterized function, their gene ID is shown.

Overall, the PPI diagrams Fig 5A and 5B proposed that both PvSRS5 and PvSRS6 TF can interact with NAC proteins, which are key regulators of plant growth, development, and response to abiotic stress [54] and with auxin response factors (ARFs) and auxin-induced proteins Fig 5A and 5B. ARFs are plant-specific TF that regulate early auxin-responsive genes and play essential roles in plant growth, development, and phytohormone signaling [55]. In Lotus, the NF-Y/ STY signaling cascade regulates the expression of *LjYUCCA1* and *LjYUCCA11*, that encode for flavin monooxygenase-like enzymes that mediate the limiting step in tryptophan-dependent auxin biosynthesis [15]. Our results proposing interaction between PvSRS and auxing/related proteins (Fig 5) may be related to the importance of auxin signaling in the SNF, both during rhizobia infection and nodule formation [56,57].

## Discussion

In plants, the *SRS/STY/SHI* genes constitute a TF family with diverse and essential functions to regulate development of different plant organs as well as the response to different abiotic stresses [13]. Phylogenies of *SRS* genes have provided information for studies of evolution and developmental genetic pathways from plants from different families including legumes [13,15,16,18,19]. In this work we present a phylogeny of Arabidopsis and four legume species including the model legumes Medicago and Lotus as well as two very important legume crops: soybean and common bean. The two characteristic domains from the *SRS* gene family: *Znf_LRP1* and *SHI_C*, were verified in each of the 60 SRS proteins included in the phylogenetic tree. (Fig 1) highlights clades that group genes that share high expression in similar tissues: Arabidopsis roots and legume roots and nodules elicited during the SNF and likely share regulatory functions. In addition, we searched for the best hit of each *PvSRS* gene to *SRS* genes from Arabidopsis and from the three legumes included in our phylogeny, as well as for the gene expression level reported for nodules of legumes or root of Arabidopsis (S1 Fig). This analysis reveals similarities in the expression level of *SRS* putative orthologous genes. For example, the genes with best hits to *PvSRS4*, *PvSRS5, PvSRS46* and *PvSRS10* that are most highly expressed in common bean nodules also showed high to medium expression level in nodules of Lotus, soybean and Medicago as well as Arabidopsis roots, except for *LjSTY4* and *GmSRS6* that showed low expression (S1 Fig). Similar situation holds for *PvSRS7*, *PvSRS8 and PvSRS9* with low expression in common bean as well as their putative orthologous genes from soybean (*GmSRS7*, *GmSRS5 and GmSRS11)* as well as from Medicago (*Medtr4g071110* and *Medtr3g112510)* and Arabidopsis *STY1,* although best- hit genes form Lotus showed medium expression (S1 Fig).

Previous common bean transcriptomic analysis provided information about the number of *PvSRS* genes [11] from this important legume crop as well as about the expression of these genes in different plant tissues [20]. The *PvSRS* TF gene family presented a statistically significantly higher percentage of gene expression in the nodule as compared to other tissues [20]. Moreover, the relevant participation of SRS TF in nodule developmental programs of the Lotus and Medicago model legumes -which form determinant or indeterminant nodules, respectively- has been demonstrated [14,15]. On this basis, in this work, we undertook the study of the expression and transcriptional regulation of PvSRS TF in the common bean - *R. etli* N-fixing symbiosis using bioinformatic and experimental approaches.

Our results from the expression analysis of each of the 10 *PvSRS* genes in different developmental stages of roots and nodules from R*. etli*-inoculated common bean plants (Fig 2) agree to those previously reported [19], as shown in S1 Fig. As mentioned before, every *PvSRS* gene is expressed in inoculated roots and nodules at different stages of the N-fixing symbiosis (Fig 2), pointing to their participation as regulators of this relevant process. A different expression profile was observed in *PvSRS7* and *PvSRS9* genes since these showed a much higher expression level in roots from fertilized uninoculated plants than in roots/nodules of SNF plants, therefore their participation in the regulation of SNF might be less important than in root development.

The legumes *NIN* (*NODULE INCEPTION*) TF gene is considered a master regulator for the rhizobia symbiosis, since it plays an indispensable role in most stages of the symbiotic processes [58]. Among others, the *NF-Y* TF genes have been identified as NIN transcriptional targets in Medicago and Lotus [59]. The NF-Y (NUCLEAR FACTOR Y) is a heterotrimeric TF that is relevant for nodule primordia development [15,21,59]. Moreover, in Lotus it was shown that LjNF-Y TF is involved in the transcriptional activation of *LjSTY* TFs for biosynthesis and accumulation of local auxin in the cortical cells of the root [15,21] which induces the division of these cells and is an initial step in the organogenesis of the nodule [59]. In common beans, the three NF-Y subunits (NF-YA1, B7 and C1) that assemble into the NF-Y trimeric complex are upregulated during rhizobia symbiosis [45,60]. Common bean plants showing more efficient symbiosis, with higher nodule number, mainly occurring when both symbionts have the same geographical origin (Mesoamerican or Andean), exhibit elevated NF-Y expression [46,61]. In this work we proposed that similarly as in other legumes [14,15] PvSRS TF, required for rhizobia symbiosis, are part of the NIN - NF-Y regulatory cascade and so we investigated the possible NF-Y transcriptional activation of *PvSRS* genes. Results shown in Fig 3 and S4 Table support our proposition. The promoter regions of every *PvSRS* gene that are expressed during SNF, showed significantly over-represented *cis*-regulatory elements known as CCAAT_boxes, that are recognized by NF/Y TF for transcription activation (S4 Table). For *in planta* experimental validation, we used the *N. benthamiana* co-agroinfitration system [41] to visualize, through fluorescent protein gene fusion, the transcriptional activation of the *PvSRS10* gene promoter -most highly expressed during SNF- by PvNF-Y TF. For this experiment, we agro-infiltrated the PvNF-YA1 subunit, thus our positive result indicates the interaction of these subunits with heterologous NF/YB and C subunits, from *N. benthamiana* leaves, to form the heterotrimer required for transcription activation. The weak specificity for the interaction among different NF-YA, B and C subunits has been shown for Arabidopsis and Medicago NF-Y trimeric TFs [43,44,48]. Transcriptomic data has shown that different *NF-YB* and *NF-YC N. benthamiana* genes are highly expressed in mature leaves [50]. The interaction of NF-Y subunits in *N. benthamiana* heterologous system has been reported [59]. Our results clearly showed the PvNF-Y TF as a transcriptional activator of *PvSRS10* gene (Fig 3).

We attempted to identify another transcription regulator of *PvSRS10* gene. Focusing on PvMADS-domain/PvAGL TF was deemed relevant given the important participation of members of this gene family as positive regulators of different stages of the common bean SNF -root development, rhizobial infection, expression of early symbiotic genes, nodule formation and function, autoregulation of nodulation process- probably resulting from their potential interplay with PvNIN [22]. In this work we showed that the promoter regions of *PvSRS* genes had CArG_boxes, recognized by the AGL TF for transcriptional activation, significantly over-represented (S4 Table). Similar as for PvNF-Y TF, we demonstrated the *in planta* transcriptional activation of *PvSRS10* promoter by PvFUL-like TF (Fig 3). In addition, we showed (Fig 4) that the expression level of *PvSRS* genes is modified accordingly in transgenic nodules with modulated expression -silencing or over-expression- of *PvFUL*-like gene. On this basis, we propose PvFUL-like MADs-domain TF as a novel transcriptional activator of *PvSRS10* genes.

Studies in *L. japonicus* have revealed that auxin synthesis and accumulation pathways, such as *YUCCA1* and *YUCCA11*, which play pivotal roles in cortex cell divisions, vascular bundle formation and nodule primordia establishment [15,21] are regulated by the NIN/ NF-Y/ SRS cascade [15]. In relation to this, the PPI network diagram (Fig 5), predicted interactions of PvSRS5 and PvSRS6 TF proteins with auxin response factor (ARF) and with auxin-induce pathways proteins, something that would be important for the regulation of different stages of the SNF by this phytohormone in common bean. In addition, the PPI network diagram (Fig 5) predicted interaction of these PvSRS proteins with NAC TF proteins, known to participate in the regulation of plants responses to abiotic stresses [62,63]. This might be related to the response of *PvSRS* genes in common bean under salt stress [19].

The data presented in this work subscribes to the participation of common bean PvSRS TF in the N-fixing symbiosis with rhizobia, a relevant process for sustainable agriculture. Similar, as in the model legumes Lotus and Medicago [14,15], we found that the PvNF-Y TF targets the *PvSRS10* gene. In addition, we provide evidence for a novel transcriptional activator of *PvSRS10*, the PvFUL-like TF from the MADS-domain/ AGL TF family. We propose that the PvSRS TF role in the SNF is exerted through promoting auxin biosynthesis, similar as Lotus which also develops determinant nodules [15].

## Supporting information

S1 Fig Best hits of common bean *PvSRS* proteins in three legume species and *Arabidopsis thaliana.* The heatmap represents gene expression levels in functional legume nodules or in Arabidopsis roots, corresponding to data reported for each species. The best hits of PvSRS proteins to a SRS protein from each species and the percentage of protein identity is shown.
(TIF)

S2 Fig Controls experiments for agroinfiltration of *N. benthamiana* leaves. The red fluorescence, derived from the constitutive expression of the *tDTomato* gene present in the plasmid backbone, was observed in *N. benthamiana* leaves agroinfiltrated with the pTDTO empty vector (S2A), or each of the effector plasmids RFP_OE/NF-YA1 (S2B) or RFP_OE/FUL (S2 C). In leaves co-infiltrated with each of the effector plasmids plus the empty vector pBGWFS7, bearing the GFP without any cloned promoter (S2D, E), only red fluorescence is observed, due to the expression of TDT. Leaves infiltrated with only the empty vector pBGWFS7 (S2F) or only the reporter plasmid (S2G), showed no fluorescence, thus indicating that the *SRS10* gene promoter was not expressed by endogenous TF.
(PDF)

S1 Table Protein sequences of SRS-TFs from *P.vulgaris* (10), *L.japonicus* (9), *M.truncatula* (10), *G.max* (21) and *A.thaliana* (10), considered in the phylogenetic analysis. (XLSX)

S2 Table Primers used for qRT-PCR expression analysis. (DOCX)

S3 Table Expression levels of *PvSRS* obtained by qRT-PCR. (XLSX)

S4 Table *cis*-regulatory elements for AGL and NF-Y transcription factors binding statistically overrepresented within the 4-kb upstream promoter regions of each *PvSRS* gene. (XLSX)

S5 Table Expression level of *N. benthamiana NF-YB* and *NF-YC* genes in mature leaves. (https://nbenthamiana.jp/nbrowser/profile).
(XLSX)
