# Supplementary figures and images for: Expression profiling and transcriptional regulation of the SRS transcription factor gene family of common bean (Phaseolus vulgaris) in symbiosis with Rhizobium etli
Source: PLoS One. 2025 May 2;20(5):e0321784. doi: 10.1371/journal.pone.0321784 (PMC12047762; doi:10.1371/journal.pone.0321784)

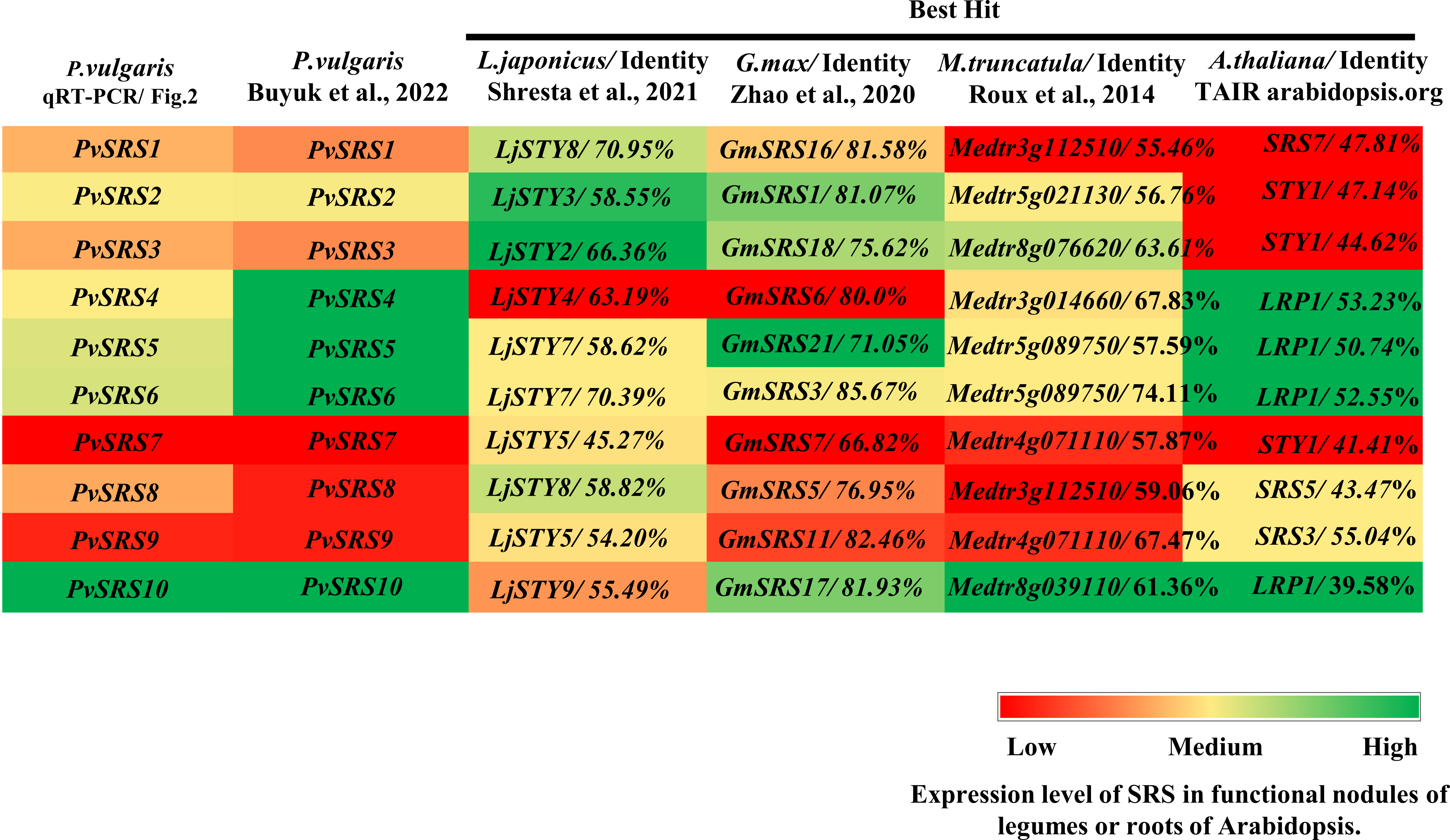

Supplement: S1 Fig — The heatmap represents gene expression levels in functional legume nodules or in Arabidopsis roots, corresponding to data reported for each species. The best hits of PvSRS proteins to a SRS protein from each species and the percentage of protein identity is shown. (TIF) [file pone.0321784.s001.tif]

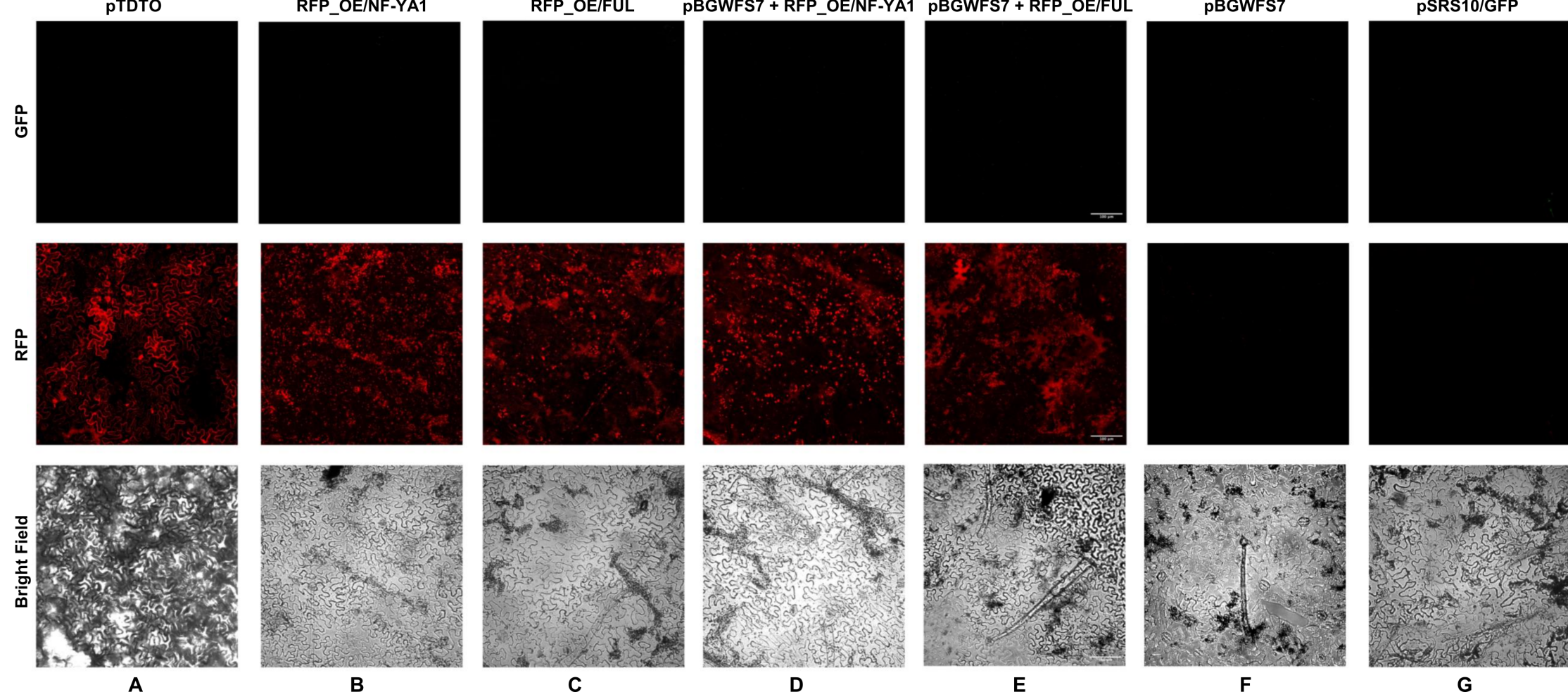

Supplement: S2 Fig — The red fluorescence, derived from the constitutive expression of the tDTomato gene present in the plasmid backbone, was observed in N. benthamiana leaves agroinfiltrated with the pTDTO empty vector (S2A), or each of the effector plasmids RFP_OE/NF-YA1 (S2B) or RFP_OE/FUL (S2 C). In leaves co-infiltrated with each of the effector plasmids plus the empty vector pBGWFS7, bearing the GFP without any cloned promoter (S2D, E), only red fluorescence is observed, due to the expression of TDT. Leaves infiltrated with only the empty vector pBGWFS7 (S2F) or only the reporter plasmid (S2G), showed no fluorescence, thus indicating that the SRS10 gene promoter was not expressed by endogenous TF. (PDF) [file pone.0321784.s002.pdf]
