# Supplementary material for: Expression profiling and transcriptional regulation of the SRS transcription factor gene family of common bean (Phaseolus vulgaris) in symbiosis with Rhizobium etli
Source: PLoS One. 2025 May 2;20(5):e0321784. doi: 10.1371/journal.pone.0321784 (PMC12047762; doi:10.1371/journal.pone.0321784)
Supplement: S2 Table — (DOCX) [file pone.0321784.s004.docx]

| **S2 Table.** Primers used for qRT-PCR expression analysis | | |
| --- | --- | --- |
| Gene ID | Forward (5´-3´) | Reverse (5´-3´) |
| Phvul.001G009800/ *PvSRS1* | CTTGCGCTCCACTTCCTATC | ACACGTGCCCTCCTATGTTC |
| Phvul.002G166700/ *PvSRS2* | CACGCCAACAATCAGAGAGA | GAGCGAAGCAACAATCACAA |
| Phvul.003G258100/ *PvSRS3* | AACATTGGGGGACATGTGTT | CCAGAAGAAGAGACAACGCC |
| Phvul.005G026300/ *PvSRS4* | AAGGGACGTGGATACGACTG | GGCATTGGAGTTGGAACTGT |
| Phvul.006G030400/ *PvSRS5* | TCAGTTATGGCAAGAGCACG | AATCCTTTTTCGCCTGGTTT |
| Phvul.008G200700/ *PvSRS6* | GGGGTGGAGAATAGGGATGT | CCAAGTAACCCTCCACCAGA |
| Phvul.009G004300/ *PvSRS7* | CCCGGTATGCCATATTTCAC | TGTTTCTAGGTGCACTTGCG |
| Phvul.009G013700/ *PvSRS8* | ACACTCAACTCGTCTCCGCT | CTGTTCATCTGAAGCGTCCA |
| Phvul.011G041200/ *PvSRS9* | TGCAAGTGCAACTATGCCTC | ATTCTCTTCTTTCCCCGCAT |
| Phvul.011G206400/ *PvSRS10* | ATCAGTGGCCATGTGTTCAA | GAACCCCAATTGCAGAAGAA |
| Phvul.006G110100/ *UBQ9* | CGCTTCCTCCTTTGTTTCGTT | ATGTATGTGGGCAACACCTAT |
| Phvul.007G273500/ *MDH* | ATCGCCTAGACCCTCTTCGT | TCAGCACCCAACATCACTCC |
| Phvul.001G039700/ *HSP* | CGGGGAGAGAAAGAGAGAAGA | AACACAAACTTCCGCATGAAC |
